# Sphingosine Kinase 1 Is Required for Mesothelioma Cell Proliferation: Role of Histone Acetylation

**DOI:** 10.1371/journal.pone.0045330

**Published:** 2012-09-17

**Authors:** Satish Kalari, Nagabhushan Moolky, Srikanth Pendyala, Evgeny V. Berdyshev, Cleo Rolle, Rajani Kanteti, Archana Kanteti, Wenli Ma, Donghong He, Aliya N. Husain, Hedy L. Kindler, Prasad Kanteti, Ravi Salgia, Viswanathan Natarajan

**Affiliations:** 1 Department of Cancer Biology, Beckman Research Institute of the City of Hope, Duarte, California, United States of America; 2 Department of Pharmacology, University of Illinois at Chicago, Chicago, Illinois, United States of America; 3 Institute for Personalized Respiratory Medicine, University of Illinois at Chicago, Chicago, Illinois, United States of America; 4 Section of Hematology/Oncology, Pritzker School of Medicine, University of Chicago, Chicago, Illinois, United States of America; 5 Pathology, Pritzker School of Medicine, University of Chicago, Chicago, Illinois, United States of America; Johns Hopkins School of Medicine, United States of America

## Abstract

**Background:**

Malignant pleural mesothelioma (MPM) is a devastating disease with an overall poor prognosis. Despite the recent advances in targeted molecular therapies, there is a clear and urgent need for the identification of novel mesothelioma targets for the development of highly efficacious therapeutics.

**Methodology/Principal Findings:**

In this study, we report that the expression of Sphingosine Kinase 1 (SphK1) protein was preferentially elevated in MPM tumor tissues (49 epithelioid and 13 sarcomatoid) compared to normal tissue (n = 13). In addition, we also observed significantly elevated levels of SphK1 and SphK2 mRNA and SphK1 protein expression in MPM cell lines such as H2691, H513 and H2461 compared to the non-malignant mesothelial Met5 cells. The underlying mechanism appears to be mediated by SphK1 induced upregulation of select gene transcription programs such as that of CBP/p300 and PCAF, two histone acetyl transferases (HAT), and the down regulation of cell cycle dependent kinase inhibitor genes such as p27Kip1 and p21Cip1. In addition, using immunoprecipitates of anti-acetylated histone antibody from SphK inhibitor, SphK-I2 treated Met5A and H2691 cell lysates, we also showed activation of other cell proliferation related genes, such as Top2A (DNA replication), AKB (chromosome remodeling and mitotic spindle formation), and suppression of p21 CIP1 and p27KIP1. The CDK2, HAT1 and MYST2 were, however, unaffected in the above study. Using SphK inhibitor and specific siRNA targeting either SphK1 or SphK2, we also unequivocally established that SphK1, but not SphK2, promotes H2691 mesothelioma cell proliferation. Using a multi-walled carbon nanotubes induced peritoneal mesothelioma mouse model, we showed that the SphK1−/− null mice exhibited significantly less inflammation and granulamatous nodules compared to their wild type counterparts.

**Conclusions/Significance:**

The lipid kinase SphK1 plays a positive and essential role in the growth and development of malignant mesothelioma and is therefore a likely therapeutic target.

## Introduction

Malignant pleural mesothelioma (MPM) is a highly aggressive and invasive neoplasm of the pleura linked with asbestos exposure in a majority of patients [1]). The incidence of MPM is anticipated to increase during the first half of this century with no effective treatment modalities other than chemotherapy, with an overall survival rate of less than 15% over 5 years [1]. Interestingly, one novel therapeutic strategy in MPM treatment is the use of inhibitors that suppress the activity of histone deacetylases (HDACs) [2], [3]. Prevention of deacetylation of histones results in the transcriptional inactivation of the associated genes and the cells undergo apoptosis. Currently, ten HDAC inhibitors are in various stages of cancer clinical trials. Only one HDAC inhibitor, suberonylanilide hydroxamic acid (SAHA), marketed as Zolinza (vorinostat) has been approved by US Foods and Drugs Administration (FDA) for the treatment of cutaneous T-cell lymphoma (http://www.cancer.gov/cancertopics/druginfo/fda-vorinostat) [4]. It is currently being evaluated in Phase III clinical trials in MPM. In order to make a significant impact on the overall survival of MPM patients, newer molecular mechanisms need to be identified and targeted for the development of highly efficacious therapies.

Sphingosine kinase (SphK) is a lipid kinase that phosphorylates sphingosine to sphingosine-1-phosphate (S1P) and mammals express two functional SphK isoenzymes, SphK1 and SphK2. S1P, generated intracellularly either by SphK1 or SphK2, is transported out of the cells where it acts as ligand for five G protein coupled S1P_1–5_ receptors and regulates several vital cellular processes such as growth and differentiation, survival, cytoskeletal rearrangements and motility, angiogenesis, and immune defense [5]. It also acts intracellularly to regulate calcium homeostasis (6), cell growth and suppression of apoptosis [7]–[12] and cell motility [13].

A variety of stimuli including growth factors and cytokines activate SphK1; however, activation of SphK2 is unclear. SphK1 has been identified as a potential therapeutic target in cancer [14]–[18] as evidenced by two lines of investigations: (i) overexpression of Sphk1 in fibroblasts resulted in the acquisition of transformed phenotype and (ii) MCF7 cell xenografts over-expressing Sphk1 grew more rapidly in nude mice [19]. Furthermore, SphK1 mRNA was significantly elevated in various tumor tissues (brain, breast, lung, ovary, stomach, colon) [17], and a higher expression of SphK1 in human astrocytoma tissue correlated with a shorter patient survival time [20]. Overexpression of SphK1 offered protection to tumor tissues against anticancer drugs by shifting the ceramide/S1P balance towards the cytoprotective S1P [21]–[23] and also by the inhibition of cytochrome c release from mitochondria induced by chemotherapeutic agents [24]. As there are no known forms of oncogenic mutations of Sphk1, by definition it is not an oncogene; however it demonstrates all the attributes of an oncogene such as colony growth in soft agar and foci formation as well as promoting cell transformation with Ras [17]. A 2-fold increase of SphK1 mRNA expression and overwhelmingly positive immunostaining for SphK1, as compared to with patient-matched normal tissue, was observed in lung cancer tissues [25]. While there is increasing evidence to suggest a role for SphK1 and S1P in breast and ovarian cancer [27]), very little is known on the role of SphK2 in tumorigenesis. SphK2 has been reported to suppress growth and enhance apoptosis [27], [28].

While investigating the potential mechanisms underlying mesothelioma cell proliferation, we noted a significant increase in mRNA and protein expression of SphK1 in mesothelioma cell lines compared to control cell line, and tumor tissues exhibited relatively high levels of SphK1 protein. Given the positive role of SphK1 in cancer, we hypothesized that high expression of SphK1 may play an important role in the development of mesothelioma. In the current study, we define the functional consequence of the down-regulation of SphK1 on the growth of mesothelioma cells and investigated the potential link between SphK1 and acetylation of H3 and H4 histones on mesothelioma cell proliferation. Our results indicated that SphK1, but not SphK2, regulates mesothelioma cell proliferation through histone acetylation signal transduction. These studies provide novel insights into SphK1 dependent modulation of histone acetylation through histone acetyltransferases and/or histone deacetylases in regulating mesothelioma cell proliferation.

## Experimental Procedures

### Cell Lines and Tissue Specimens

H2691 (epithelioid), H2461 (epithelioid), H513 (epithelioid), H2596 (sarcomatoid), H2373 (sarcomatoid), and non-malignant mesothelial cells (Met5A) were obtained from the American Type Culture Collection. Met5A were cultured in Medium 199 containing 10% fetal bovine serum (FBS) plus 1% Penicillin/Streptomycin antibiotic while the mesothelioma cell lines were cultured in RPMI 1640 medium containing 10% FBS and 1% Penicillin/Streptomycin. The Institutional Review Board at the University of Chicago has approved use of human materials in this study for Dr. Ravi Salgia University of Chicago, Chicago).

### Cell Proliferation Assay

Non-malignant and malignant mesothelioma cell lines seeded at a density of 1×10^4^ cells/well in 96 well plate were allowed to grow for 24 h in RPMI-1640 medium containing 10% FBS. Cells were starved overnight (18 h) in serum-free RPMI-1640 medium and challenged with 5% serum or pretreated with inhibitors for 24 h prior to serum challenge for 24–48 h. During the last 1 h of stimulation, MTS was added as per manufacturer’s instruction and water soluble reddish brown formazan adduct produced was measured at 490 nm with an ELISA plate reader.

**Table 1:** Forward and reverse primers used for specific gene amplification in the SphK1 ChIP assay.

Gene	Forward Primer	Reverse Primer
**AKB**	GTCAGCACACTAGCCCCAAT	GGGAGAGTAGCAGTGCCTTG
**TOP2A**	GTGACACTTCCATGGTGACG	TGGTTCTTCTGGACGGAGAC
**P27KIP1**	GCCATATTGGGCCACTAAAA	CTCCGCTGATCAAATGGACT
**P21CIP1**	GCAGAGAGGTGCATCGTTTT	GACACATTTCCCCACGAAGT
**CDK2**	ACAAGTTGACGGGAGAGGTG	GAAGTCCCTCCCTGCTCTCT
**HAT1**	CAGGGAAAAGCGTCATGATT	TGCGCTAGCTCAGAAGTTGA
**MYST2**	CGAAATTTTGCCCAAGGTAA	TGGGAAGTTCGATCCTTTTG
**GAPDH.**	TACTAGCGGTTTTACGGGCG	TCGAACAGGAGGAGCAGAGAGCGA

### RNA Isolation, Reverse Transcription and Quantitative Real-time-PCR

Total RNA was isolated from Met5A and mesothelioma cell lines using TRIzol® reagent (Invitrogen, Carlsbad, CA) according to the manufacturer’s instructions, and RNA was quantified by spectrophotometer. cDNA was prepared using the iScript cDNA synthesis kit (Bio-Rad, Hercules, CA). Quantitative PCR was performed to assess expression of SphK1, SphK2, 18S along with several other cell cycle relevant genes as mentioned below using primers designed based on human mRNA sequences. SphK1 primers: forward 5′ATGCTGGCTATGAGCAGGTC3′ and reverse 5′GTGCAGAGACAGCAGGTTCA3′; SphK2 primers: forward 5′CCCGGTTGCTTCTATTGGT3′ and reverse 5′GACAGCCCAGCTTCAGAGAT3′; p300/CBP-associated factor (PCAF): forward 5′ ACACACAGCCACGCTGATAA3′ and reverse 5′CAAACCCTGGTGGGAATTTA3′; CBP: forward 5′ACTGGCAGACCTCGAAAAGA3′ and reverse 5′GCCGCAAAAATTTGTTCACT3′; MYST2/HBO1: forward 5′TACACTGCAGACGCTGGTTC3′ and reverse 5′GCCGCAAAAATTTGTTCACT3′; p27Kip1/CDKN1B: forward 5′AGGTGCTTGGGAGTTTTGAA3′ and reverse 5′TGTTTACACAGCCCGAAGTG3′; p21Cip1/CDKN1A: forward 5′GATTAGCAGCGGAACAAGGA3′ and reverse CAACTACTCCCAGCCCCATA3′ and 18 S primers: forward 5′GTAACCCGTTGAACCCCATT 3′ and reverse 5′CCATCCAATCGGTAGTAGCG3′. Real-time PCR was performed using iQ SYBR Green Supermix and the iCycler real-time PCR detection system (Bio-Rad, Hercules, CA). Amplicon expression in each sample was normalized to its 18S RNA content. The relative abundance of target mRNA in each sample was calculated as 2 raised to the negative of its threshold cycle value times 10^6^ after being normalized to the abundance of its corresponding 18S, e.g.{ [2^-(IL-8 threshold cycle)^/2^-(18 S threshold cycle)^]×10^6^}.

### Transfection of Cells with siRNA or shRNA

Met5A and mesothelioma cell lines grown to ∼50% confluence in 6-well plates were transfected with Gene Silencer® (Gene Therapy System, San Diego, CA) transfecting agent with scrambled, SphK1or SphK2 siRNA (50 nM, Santa Cruz Biotechnology, Santa Cruz, CA). GFP/SphK1-shRNA (2 µg) or non-silencing-GIPZ shRNAmir control (2 µg, Open Biosystems Products, Huntsville, AL) were transfected using FuGene HD (3 µl, Roche Applied Science, Indianapolis, IN) in serum-free medium according to the manufacturer’s recommendation. After 3 h post-transfection, 1 ml of fresh complete Medium 199 containing 10% FBS for Met5A or RPMI1640 with 10% FBS for mesothelioma cell lines was added and cells were cultured for an additional 48 h for analysis of SphK1 and SphK2 by Western blotting.

### Preparation of Cell Lysates and Immunoblotting

The cell lysates were prepared as described [13] and the protein concentrations determined using BCA protein assay kit (Thermoscientific, Rockford, IL) using bovine serum albumin as the standard. Equal amounts of protein (20–30 µg) were subjected to 10% or 12% SDS-PAGE gels, transferred to polyvinylidene difluoride membranes, blocked and incubated with primary, and followed by secondary antibodies [13]. The membranes were developed with enhanced chemiluminescence detection system according to the manufacturer’s instructions.

### Immunohistochemistry and Tissue Microarrays

Sixty two MPM (49 epithelioid and 13 sarcomatoid) and 13 normal tissue samples were processed into a tissue microarray (TMA) under an institutional review board approved protocol. The controls were generated from uninvolved lung and pleura tissues with normal lung parenchyma, fibrotic pleura, giant cell reaction, and reactive mesothelioma morphologies. The TMA sections were deparaffinized and subjected to antigen recovery as described [29]. Non specific binding was blocked with BSA and then subjected to immunostaining using anti-SphK1 antibody (rabbit polyclonal; Abcam, Cambridge, UK), followed by goat anti- anti-rabbit IgG conjugated to a horseradish peroxidase–labeled polymer (Envision+ System, DAKO, Carpinteria, CA). The visualization was carried out using diaminobenzidine chromogen (DAKO), and then counterstained with hematoxylin. Appropriate negative controls for the immunostaining were prepared by omitting the primary antibody step and substituting it with non-immune mouse or rabbit serum. The stained slides were scored on a modified procedure previously described [29]. The relative intensity as well as %positive cells (IOD) for each sample was scored on a scale of 0–3 and the two scores were then multiplied to obtain the final score (scale; 0–9).

### Measurement of Intracellular [^32^P]S1P

Control or mesothelioma cells grown in 35-mm dishes to ∼90% confluence were labeled with [^32^P]orthophosphate (20 µC_i_) in phosphate-free Dulbecco’s modified Eagle’s medium without serum for 3 h. The media was aspirated, and cells were challenged with 1 ml of minimal Eagle’s medium alone or media containing sphingosine (1 µM) in the presence of 0.1% BSA for 60 min. Lipids were extracted under acidic condition by addition of 2 ml of methanol : HCl (100 : 1 V/V), cells were harvested with a cell scraper and total lipids extracted by addition of 2 ml of chloroform and 0.8 ml of 1N HCl to give a final ratio of 1:1:0.9 of chloroform:methanol:acidic aqueous phase. [^32^P]S1P generated in cells was separated from the total lipid extracts by thin layer chromatography as previously described [30], [31]. Radioactivity in S1P was determined by liquid scintillation counting and data were normalized to 10^5^ cells.

**Figure 1:**
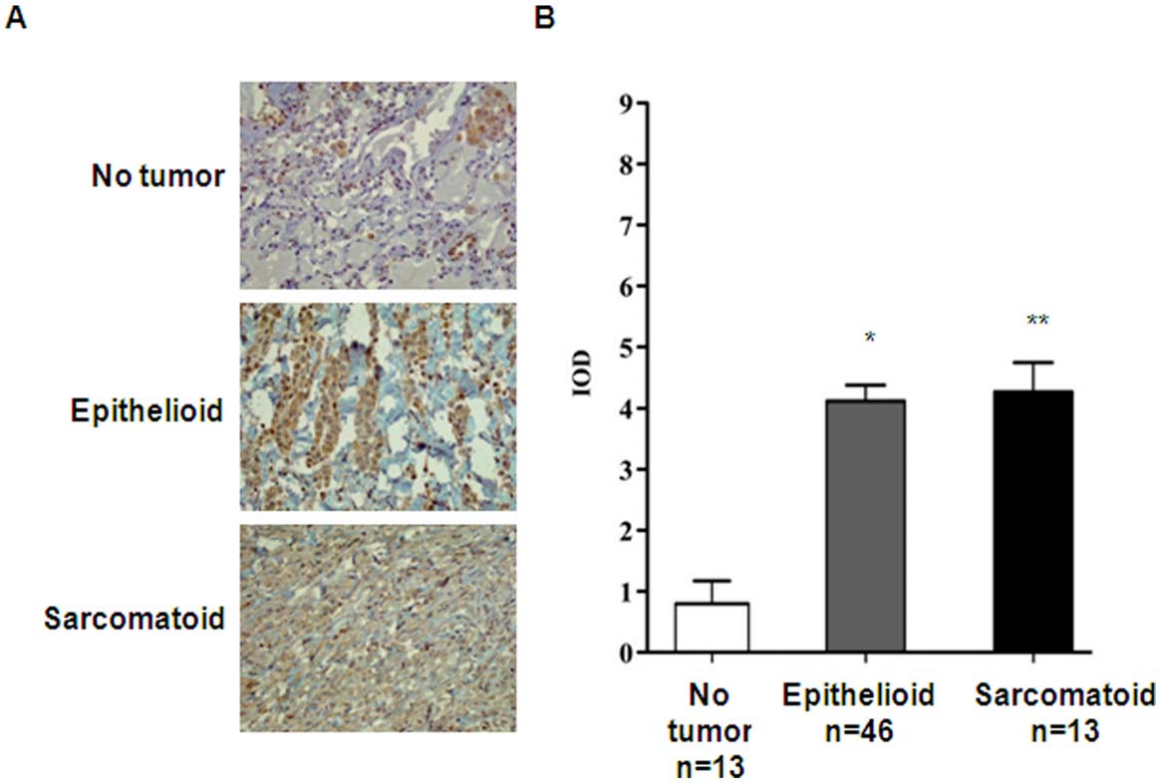
Expression of SpHK1 in malignant pleural mesothelioma tumor tissues. (A) Non-tumor, mesothelioma epithelioid and sarcomatoid tissue microarray specimens were immunostained with SphK1 antibody as described under “Experimental Procedures” Shown is a representative image from several immunostained protein microarrays (Non-tumor: n = 13; Epithelioid malignant pleural mesothelioma: n = 49; Sarcomatoid malignant pleural mesothelioma: n = 13; ‘n’ represents the number of patients). (B) Images from (A) were analyzed and expression of SphK1 in non-tumor, epithelioid and sarcomatoid malignant pleural mesothelioma semi-quantified by Image analyzer. The y-axis represents expression of SphK1 as a product of relative intensity multiplied by % of cells positive (IOD) each on a scale of 0–3.

### Pretreatment of Cells with SphK Inhibitor, SphK-I_2_

Non-malignant or malignant mesothelioma cells grown to ∼90% confluence were pre-incubated with SphK-I_2_ (2-(p-hydroxyanilino)-4-(p-chlorophenyl) thiazole (Cayman Chemical, Ann Arbor, MI)) (13) (1–10 µM) in serum-free or media containing 1% FBS as indicated for 24 h prior to stimulation with serum or other agonist.

**Figure 2:**
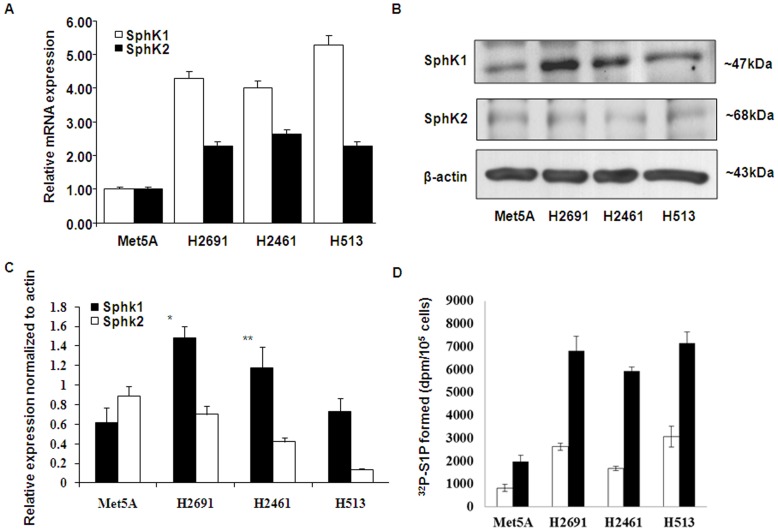
Expression and generation of S1P in control and mesothelioma cell lines. (A) Non-malignant Met5A and malignant mesothelioma cell lines H2691, H2461 and H513 were cultured to ∼90% confluence in 35-mm dishes. Total RNA was isolated, and mRNA expression of SphK1 and SphK2 was determined by real-time RT-PCR and normalized to 18S and expressed relative to Met5A expressed levels. Values are mean ± SEM of six independent experiments. (B) Cell lysates (20 µg protein) from non-malignant Met5A and malignant mesothelioma cell lines were subjected to SDS-PAGE and Western blots were probed with anti-SphK1, anti-SphK2 and anti-actin (loading control) antibodies. Shown is a representative Western blot from six independent experiments. Based on molecular weight markers, on SDS-PAGE immunostaining of SphK1 (∼47 KDa), SphK2 (∼68 KDa) and β-actin (∼43 Kda) was observed. (C) Western blots from (B) were scanned and quantified using Image analyzer. (D) Confluent Met5A or mesothelioma cell lines H2691, H2461 and H513 were labeled with [^32^P] orthophosphate (20 µC_i_/ml) in DMEM-phosphate free medium for 3 h, radioactive medium was aspirated, cells were rinsed with medium and challenged with medium alone or medium containing sphingosine (1 µM) complexed to 0.1% BSA for 60 min. Lipids were extracted, separated by thin-layer chromatography and radioactivity associated with S1P was determined by liquid scintillation counting. Values are mean ± SEM of three independent experiments in triplicate and normalized to total radioactivity in the lipid extract.

### Quantification of S1P Levels by LC-MS/MS

Total cellular lipids were extracted under acidic condition using 0.1 M HCl by a modified Bligh and Dyer procedure with C17-S1P (40 pmol) that was added as an internal standard during the lipid extraction protocol. The lipid extracts were dissolved in ethanol and aliquots were analyzed for total lipid phosphorus and subjected to LC-MS/MS for quantification of S1P [30]–[32].

### Chromatin Immunoprecipitation (ChIP) Assay

Non-malignant (Met5A) and malignant mesothelioma (H2691) cell lines were grown to ∼90% and after the addition of either SphK-I_2_ (2-(p-hydroxyanilino)-4-(p-chlorophenyl) thiazole or DMSO to the cells for 24 h, they were then treated with formaldehyde. ChIP assay was performed using EZ-ChIP Kit (Upstate, Billerica, MA) and anti-acetylated histone antibody (Cell Signaling Technologies, Danvers, MA). Acetylation enriched DNA was the subjected to PCR amplification of proximal promoter using gene specific primers. As summarized in **Table 1**, the primer sets used for each gene investigated are shown.

**Figure 3:**
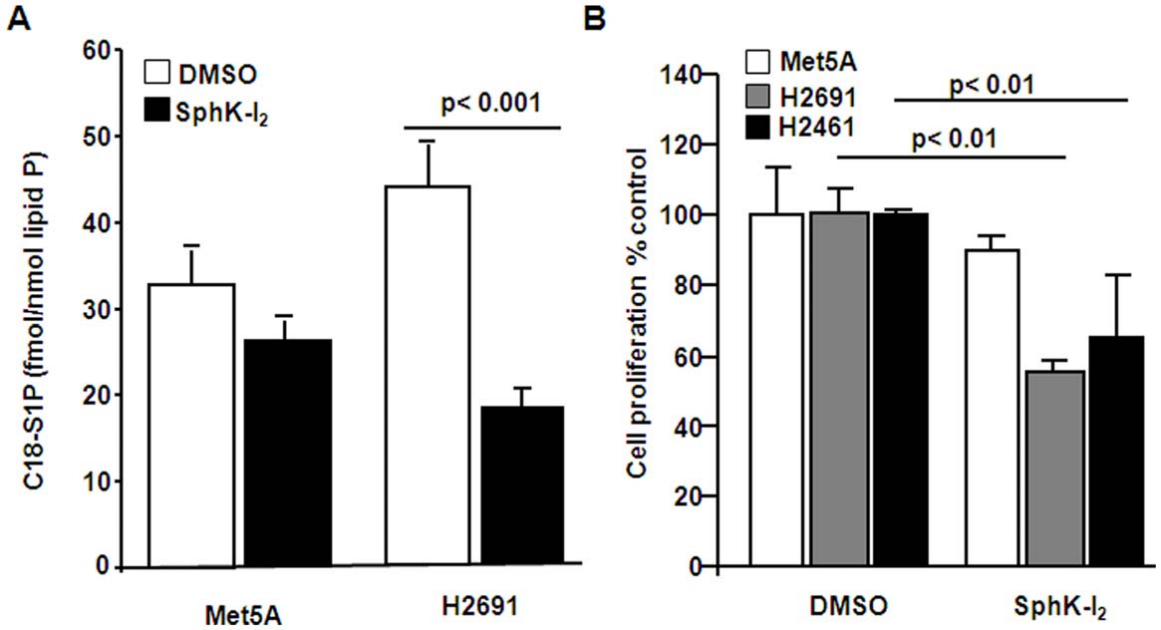
Effect of SphK inhibitor, SphK-I_2_ on intracellular S1P generation and mesothelioma cell proliferation. (A) Confluent Met5A or mesothelioma cell line H2691 (∼90% confluence) in DMEM containing 2% fetal bovine serum was pre-treated with SphK inhibitor, SphK-I_2_ (10 µM in DMSO solvent) for 24 h. Accumulation of intracellular C18-S1P was quantified by LC-MS/MS as described under “Experimental Procedures”. (B) Non-malignant Met5A or malignant mesothelioma cell lines H2691 and H2461 (3×10^3^) cells were seeded in 96-well plate in complete medium for 48 h. At the end of 48 h, cells were starved overnight in 1% FBS, treated with SphK-I_2_ (10 µM) in medium containing 1% FBS for 3 h and stimulated with medium containing 5% FBS for 16 h. Cell proliferation was evaluated using MTS assay as described under “Experimental Procedures”. Values are from three independent experiments expressed as % cell proliferation. Cell proliferation in the absence of SphK inhibitor was considered 100%. SphK-I_2_ treatment significantly attenuated serum-induced proliferation of mesothelioma cell lines H2691 and H2461 but not non-malignant Met5A cells.

**Figure 4:**
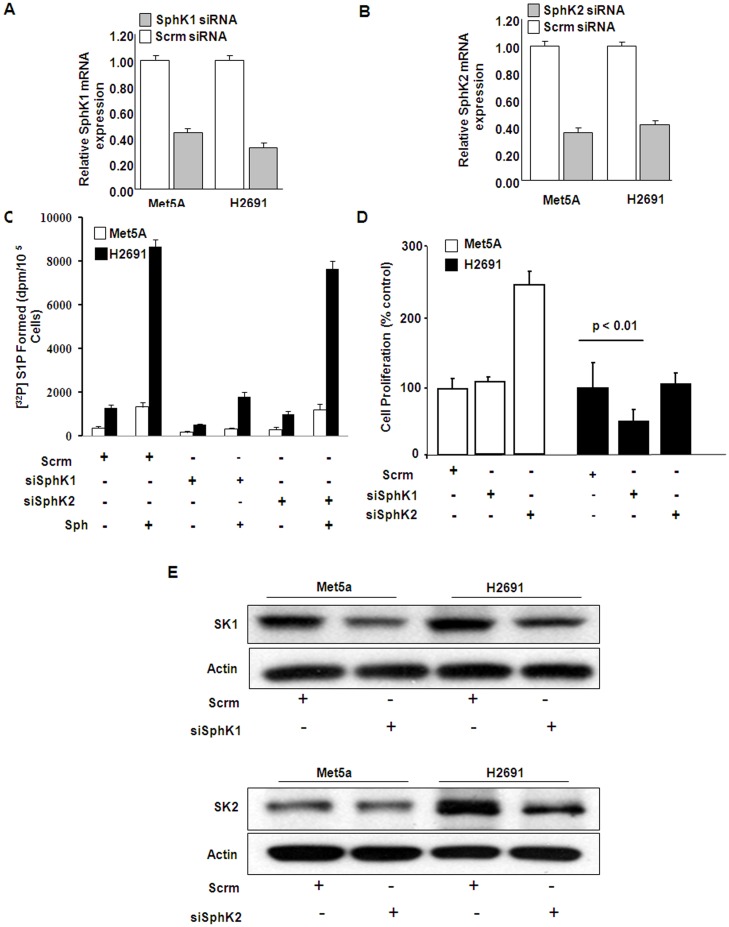
Effect of down-regulation of SphK1 and SphK2 with siRNA on mesothelioma cell proliferation. Non-malignant Met5A and malignant mesothelioma H2691 cells (∼50% confluence) were transfected with scrambled, SphK1 or SphK2 siRNA (100 nM) for 72 h as described under “Experimental Procedures”. (A) and (B), total RNA was isolated, and mRNA expression of SphK1, and SphK2 was evaluated by real-time RT-PCR and normalized to 18S. Values are the averages of three-independent experiments. (C), SphK1, but not SphK2, knockdown of H2691 cells challenged with sphingosine (Sph, 5 µM) demonstrate drastically reduced capacity to produce S1P. (D), scrambled and SphK1 and SphK2 siRNA treated cells were trypsinazied and seeded onto 96 well plates (3×10^3^) cells/well in medium containing 5% FBS for 24 h, cells were starved overnight in medium containing 1% FBS prior to stimulation with medium plus 5% FBS for 16 h. Serum stimulated cell proliferation was assayed using MTS cell proliferation assay as per manufacturer’s instruction. Values are mean ± SEM of three independent experiments in triplicate. Down-regulation of SphK1, but not SphK2, significantly attenuated serum-induced proliferation of malignant mesothelioma H2691 cells and had no effect on control Met5A cell proliferation. (E), Non-malignant Met5A and malignant mesothelioma H2691 cells (∼50% confluence) were transfected with scrambled, SphK1 or SphK2 siRNA (100 nM) for 72 h as described under “Experimental Procedures”. Cell lysates (20 µg protein) from non-malignant Met5A and H2691 cell line were subjected to SDS-PAGE and Western blots were probed with anti-SphK1, anti-SphK2 and anti-actin (loading control) antibodies. Shown is a representative Western blot from three independent experiments. Based on molecular weight markers, on SDS-PAGE immunostaining of SphK1 (∼47KDa), SphK2 (∼68KDa) and β-actin (∼43Kda) was observed.

### Induction of Peritoneal Granulomas in Mice using Multiwalled Nanotubes

Age matched (8 weeks) male C57BL/6 wild type and SphK1−/− mice were intraperitoneally injected with 0.5 ml of sterile physiological saline containing 0.5% BSA, plus 50 µg/ml or 3 mg/ml of the same sonicated solution of multiwalled nanotubes [33]. The inflammatory responses were evaluated over a period of 2 weeks. Following sacrifice, the peritoneal cavity was exposed. The diaphragm was carefully removed, and sections subjected to hematoxylin and eosin staining. Serial images were taken at×100 magnification using QCapture Pro software, and granulomas quantified using Image-Pro Plus software (Media Cybernetics Inc., MD, USA).

**Figure 5:**
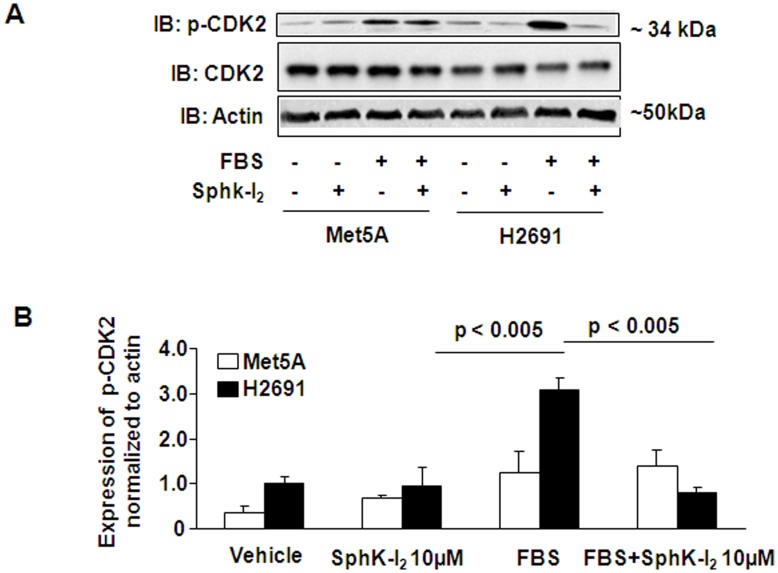
Effect of SphK inhibitor on mesothelioma entry into mitotic cell division. (A), Non-malignant Met5A and malignant mesothelioma H2691 cells (∼90% confluence) were treated with SphK inhibitor, SphK-I_2_ (10 µM), for 24 h in serum-free media prior to challenge with 5% FBS for 16 h. Cell lysates (20 µg protein) were subjected to SDS-PAGE and Western blotted with anti-phospho CDK2, anti-CDK2 and anti-actin antibodies. (B), Western blots from panel (A) were scanned and quantified by Image analyzer. Data are presented as relative ratio of phospho-CDK2 to total actin. SphK inhibitor blocked serum-induced phosphorylation of CDK2 in H2691 but not Met5A cells.

### Statistical Analyses

All results were subjected to statistical analysis using one-way analysis of variance and, whenever appropriate, analyzed by Student-Newman-Keuls test. Data are expressed as the means ± S.D. of triplicate samples from at least three independent experiments, and statistical significance was accepted at a level of P<0.05.

**Figure 6:**
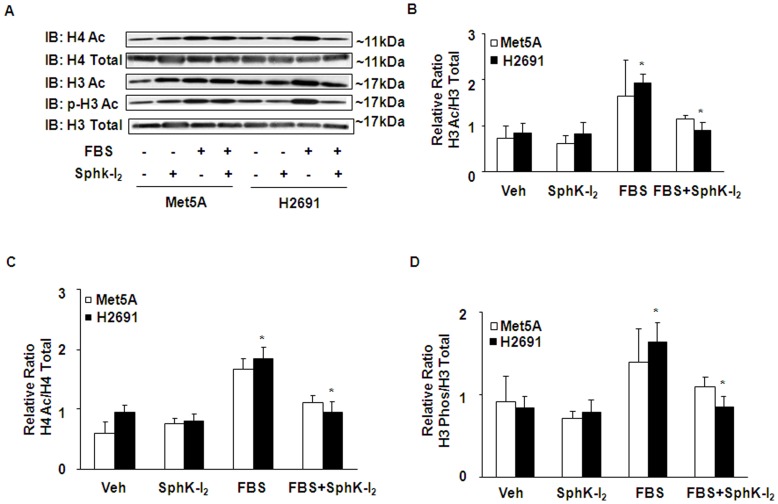
Inhibition of SphK1 modulates serum-induced histone acetylation and phosphorylation of mesothelioma cells. Met5A and H2691 cells grown to ∼90% confluence in 35-mm dishes were treated with SphK-I_2_ (10 µM) for 24 h in serum-free media as described under “Experimental Procedures”. (A), cells were challenged with media alone or media plus 5% FBS for 24 h, total cell lysates (20 µg protein) were separated by SDS-PAGE and Western blotted with anti-acetylated H3, anti-acetylated H4, anti-H3, anti-H4 and anti-phospho-H3 antibodies. Shown is a representative Western blot from three independent experiments. (B–D), Results of serum-induced acetylation of H3 and H4 and phosphorylation of H3 were analyzed by densitometry. The values are mean ± SEM compiled from three independent experiments and normalized to total H3 or H4 in the cell lysates.

## Results

### Expression of SphK1 in Archival Mesothelioma Tumor Tissues

Since SphK1 is known to be involved in tumorigenesis, the expression of both SphK1 in normal (n = 13), and mesothelioma tumor tissues (46 epithelioid and 13 sarcomatoid) was determined. As shown in **Fig. 1A and 1B**, the expression of SphK1 was significantly higher (>4 fold) in mesothelioma tumors of epithelioid and sarcomatoid origin in contrast to weaker expression in normal tissue samples. Unfortunately, due to background problems we could not get definitive answers regarding expression levels of SphK2 from archival mesothelioma tumor tissue sections.

**Figure 7:**
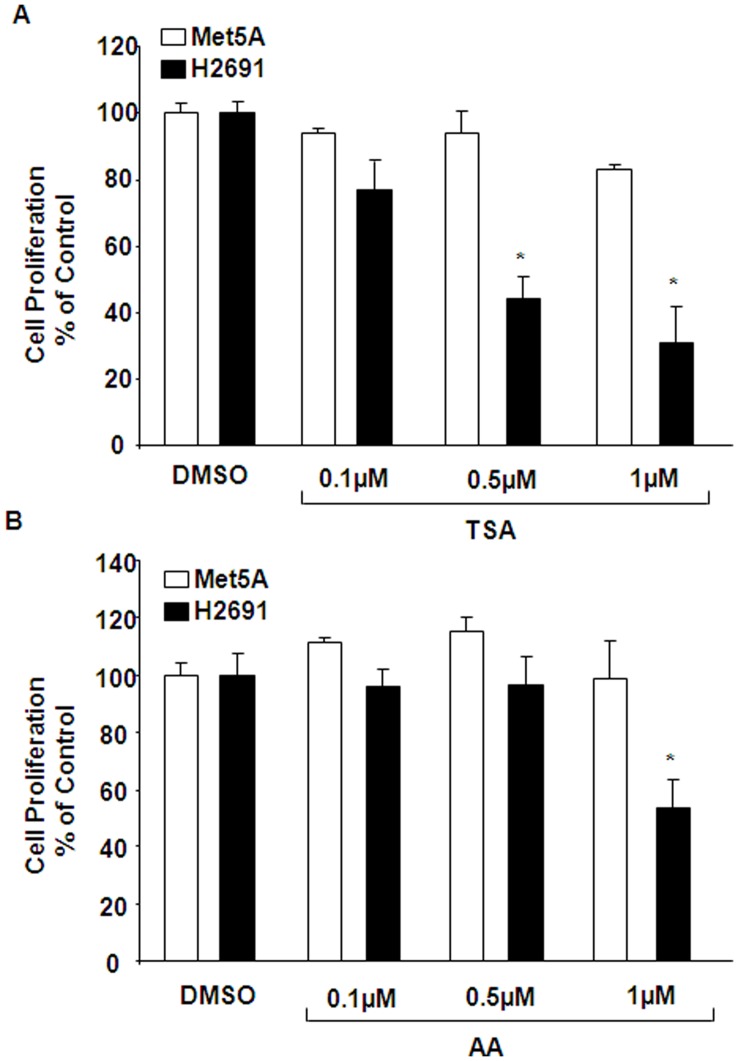
Effect of HAT and HDAC inhibitors on serum-induced proliferation of mesothelioma cells. Met5A and H2691 cells grown to ∼90% confluence in 96 well plates were exposed to different concentrations of trichostin A (0.1–1.0 µM; HDAC inhibitor) (A) or anacardic acid (10–30 µM; HAT inhibitor) (B) for 24 h prior to stimulation with 5% FBS. The yield of formazan was measured at 490 nm after MTS for 4 h. The values shown are mean ± SEM from three independent experiments and normalized to % control.

**Figure 8:**
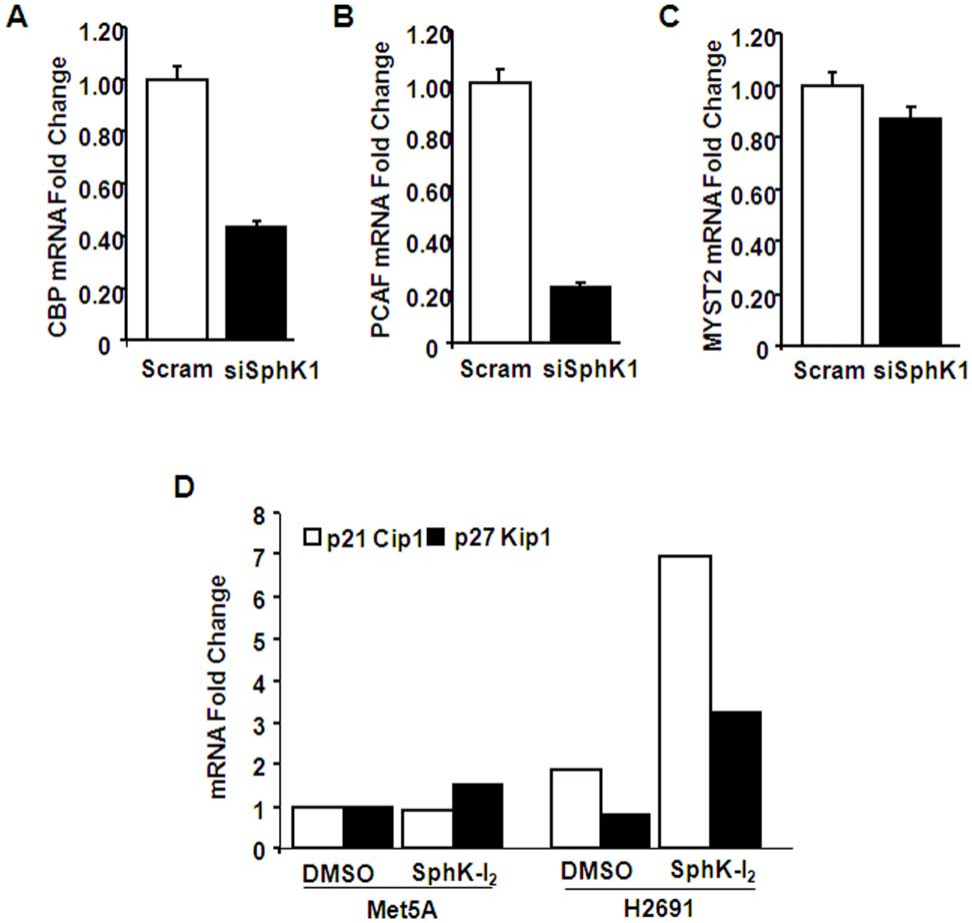
SphK1 siRNA modulates mRNA expression of CBP and PCAF, p21Cip1 and p27Kip1, but not MYST2, in mesothelioma cells. H2691 mesothelioma cells grown to ∼60% confluence in 35-mm dishes were transfected with scrambled or SphK1 siRNA (100 nM) for 48 h. Total RNA was isolated from scrambled and SphK1 siRNA-transfected cells, and real-time RT-PCR was performed in a Light Cycler using SYBR Green QuantiTect to determine mRNA levels of CBP (A), PCAF (B), MYST2 (C) p21Cip1 and p27Kip1 (D). Values are the mean ± SEM from three independent experiments normalized to 18S mRNA, and presented as fold change over 18S.

### SphK1, but not SphK2, Levels are Elevated in Mesothelioma Cells

The mRNA and protein expression profiles of SphK1 and SphK2 were examined in one control (Met5A, non-malignant transformed mesothelioma cells) and three mesothelioma cell lines using real time RT-PCR and immunoblotting with anti-SphK1 and anti-SphK2 polyclonal antibodies. Relative levels SphK1 and SphK2 transcripts normalized to 18S were significantly higher in mesothelioma cells lines (H2691, H2461, H513) compared to Met5A. The average increase in SphK1 transcripts was about 4–5 fold and SphK2 was 2 to 2.5 fold. Interestingly, SphK1 transcripts were much more elevated than Sphk2 and were at least 2 fold higher (**Fig. 2A**). Analyses of whole cell lysates by Western blots revealed that SphK1 protein expression was elevated in the mesothelioma cell lines (∼2 to 4 fold) compared to Met5A. In contrast, despite the elevated levels of Sphk2 transcripts, the protein levels were, however, much lower in mesothelioma cell lines compared to of Met5A (**Fig. 2B and 2C**). Having established a role for SphK1 in mesothelioma cell proliferation, we then investigated if serum stimulated S1P generation in mesothelioma cells. Met5A and mesothelioma cell lines were labeled with [^32^P]orthophosphate, and cultured in media containing 1% FBS in the presence or absence of exogenous sphingosine, the substrate for SphK1 and SphK2 [30]. As shown in **Fig. 2D**, both Met5A and mesothelioma cell lines demonstrated the ability to generate [^32^P]S1P from exogenous sphingosine; however, mesothelioma cell lines exhibited a higher capacity compared to the control Met5A cells. Interestingly, mesothelioma cell lines H2691 and H513 generated higher labeled S1P (∼3 fold) as compared to Met5A cells even in the absence of exogenous addition of sphingosine. These results clearly demonstrated an intrinsic higher capacity to generate S1P in mesothelioma cells that is best reflected in the absence of exogenous substrate and also supported by the relatively higher levels of SphK1 as shown in **Fig. 2B**. These results show increased protein expression of SphK1, but not SphK2 and higher S1P generation in mesothelioma cell lines.

**Figure 9:**
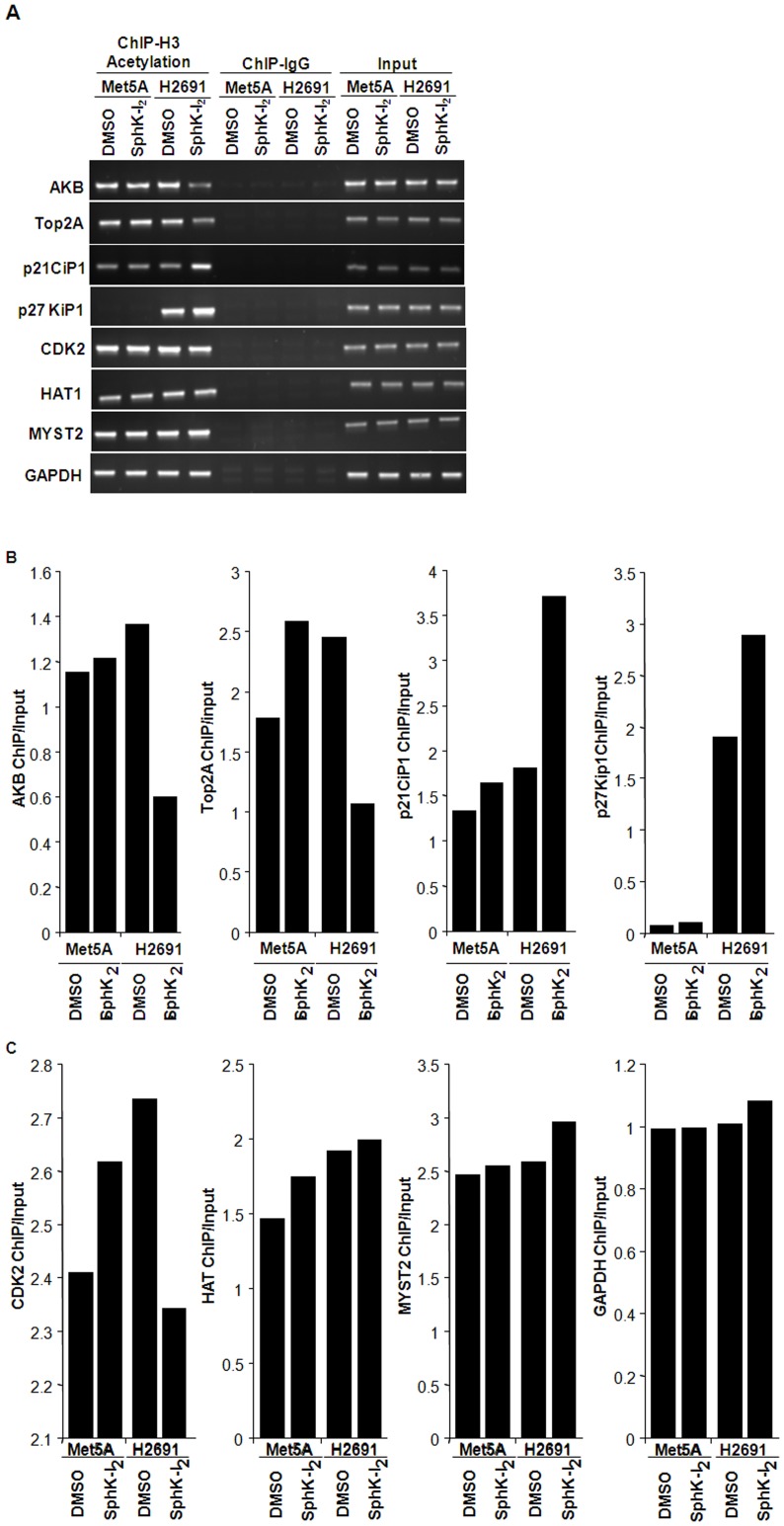
SphK1 inhibition modulates expression of chromatin remodeling and cell cycle genes through promoter acetylation. Met5A and H2691 cells grown to ∼90% confluence in 100-mm dishes were treated with SphK-I_2_ (10 µM) in RPMI-1640 medium containing 1% FBS for 24 h. Cells were fixed in paraformaldehyde and chromatin immunoprecipitation assay (ChIP) was performed with EZ ChIP (Upstate) as per manufacturer’s instructions. After immunoprecipitation with anti- pan acetylated lysine antibody, immunoprecipitated DNA was subjected to real-time PCR to quantify Aurora kinase B (AKB), Top2A, p21CiP1, p27KiP1, CDK2, HAT1, and MYST2 proximal promoter regions. GAPDH and IgG served as internal controls for loading and specificity of ChIP assay, respectively. Experiments were carried out in triplicate and a representative gel image is shown. (B and C), Images from (A) were analyzed by Image analyzer and normalized to GAPDH input. Values are averages of three ChIP assays.

**Figure 10:**
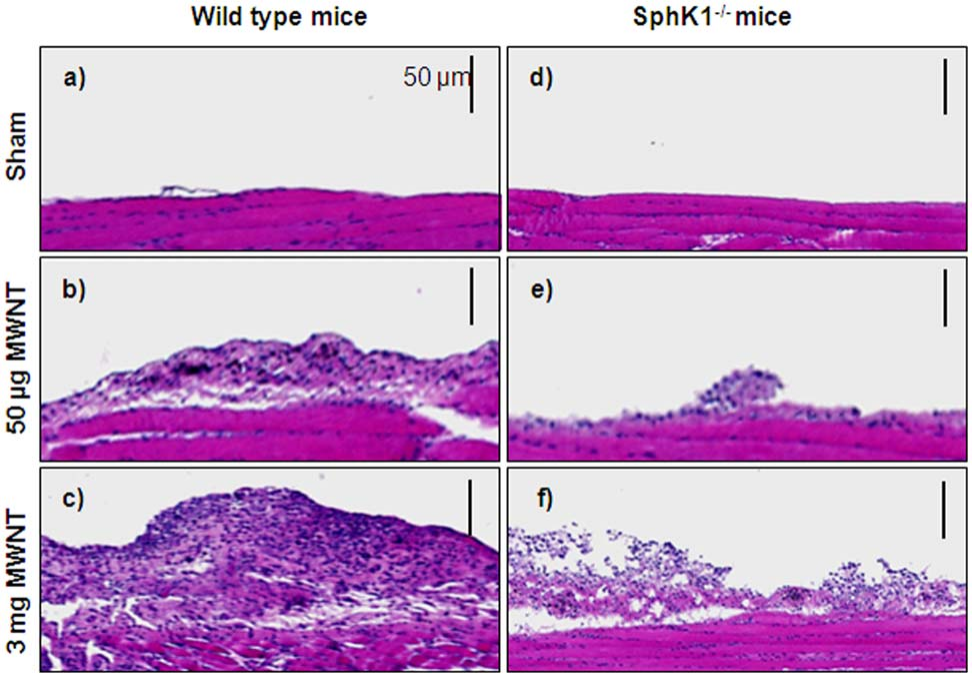
Deletion of *SphK1* gene reduces multi-walled nanotubes-induced granulamatous inflammation in mice. 8 weeks old male C57BL6/J wild type and *SphK1^−/−^* null mice (n = 6 in each group) in the same background were intraperitoneally injected with either sterile PBS or PBS containing 50 µg or 3 mg of MWNT as described under “Experimental Procedures”. After 12 days, mice were anesthetized, diaphragms were dissected, fixed with methanol/chloroform/glacial acetic acid and paraffin-embedded sections were stained with hematoxylin/Eosin. (A), Diaphragms from sham operated wild type and *SphK1^−/−^* mice show no granulamatous inflammation. (a and d), C57BL6/J mice were exposed to 50 µg or 3 mg MWNT show the presence of granulamatous inflammation on diaphragms. (b and c), *SphK1^−/−^* null mice exhibit reduced granulamatous inflammation. (e and f).

### Inhibition of SphK1 or Abrogation of its Expression Attenuates Proliferation of Mesothelioma Cells

The endogenous role of SphKs on control (Met5A) and mesothelioma (H2691 and H2461) cell proliferation was investigated using a commercially available inhibitor of SphK1 and SphK2, namely SphK-I_2_. As shown in **Fig. 3A**, pretreatment of mesothelioma cell line H2691, but not Met5A, with the inhibitor SPHK-I_2_ blocked SphK activity as reflected by the measured intracellular levels of S1P generated using LC/MS/MS [30]–[32]. Prior treatment of cells with 10 µM of SPHK-I_2_ for 24 h resulted in a significant reduction in serum-induced proliferation of mesothelioma cells (by about 40–50%); however, the control cell line Met5A was insensitive to the inhibitor at the concentration tested (**Fig. 3B**). We also knocked down protein expression of SphK1 and SphK2 with SphK1 and SphK2 specific siRNA in Met5A and H2691 mesothelioma cell lines. The mRNA (**Fig. 4A and 4B**) and protein (**Fig. 4E**) expressions of SphK1 and SphK2 were decreased by ∼80% and ∼70% after transfection with specific siRNA. We also tested the effects of SphK1 and SphK2 downregulation on generation of S1P in control Met5A and mesothelioma H2691cells. As shown in **Fig. 4C**, there was a modest increase in [^32 ^P]S1P produced when cells were transfected with scRNA; however both the cell lines, in the presence of 5 µM sphingosine (Sph), generated significantly higher amounts of [^32 ^P]S1P. In comparison, siSphK1 transduced cells when treated with Sph (5 µM) did not reveal any increase in [^32 ^P]S1P generation and the increased production of S1P in presence of sphingosine was only inhibited by siSphK1 but not siSphK2 (**Fig.** **4D**). Finally, siSphK1 transduced cells showed much reduced serum-dependent proliferation of H2691 without affecting Met5A (100 vs. 40%) while down-regulation of SphK2 had no effect on serum-mediated proliferation of H2691 (**Fig. 4E**). The control cells, however, more than doubled in number (100 vs. 250%). These results suggest a role for SphK1, but not SphK2, in serum-mediated mesothelioma cell proliferation.

### SphK Inhibitor, SphK-I_2_ Modulates Mesothelioma Cell Entry into Mitotic Cell Division

To further understand the role of SphK1 in mesothelioma cell proliferation, we next treated the cells with SphK inhibitor and determined the effect on phosphorylation of cyclin dependent kinase 2 (CDK2), which is essential for eukaryotic cell cycle G1/S phase transition. As shown in **Fig. 5A and 5B** **,** pre-treatment with the SphK-I_2_ (10 µM) for 24 h, attenuated the phosphorylation of CDK2 at Thr_160_ in H2691, but not in Met5A, as determined by immunoblotting using specific anti-CDK2 antibody. These results suggest that SphK1 regulates mesothelioma cell proliferation by facilitating the crossover of the G1/S check point in mesothelioma cell cycle and such an effect was not apparent in control Met5A cells.

### SphK Inhibitor, SphK-I_2,_ Modulates Serum-induced Histone (H) Acetylation and Phosphorylation of H3 Histone in Mesothelioma Cells

S1P and SphK2 are known to target Histone acetylation [34]. The role of SphKs in serum-mediated histone acetylation was investigated using serum starved Met5A and H2691 cells. Cells were pretreated with SphK inhibitor, SphK-I_2_ (10 µM), for 24 h prior to exposure to 5% fetal bovine serum. As shown in **Fig. 6** **A–D**, SphK-I_2_ attenuated serum-induced H3 and H4 acetylation and phosphorylation of H3 at Ser10 in H2691 but not in Met5A control cells.

### HAT and HDAC Inhibitors Attenuate Serum-induced Proliferation of Mesothelioma Cells

As SphK1 inhibition modulates mesothelioma cell proliferation and histone acetylation, the effect of histone acetyl transferase (HAT) and histone deacetylase (HDAC) inhibition on serum-induced mesothelioma cell proliferation was investigated. Met5A and H2691 were serum starved and treated with different concentrations of anacardic acid (AA; HAT inhibitor) or trichostatin (TSA; HDAC inhibitor) for 3 h prior to 5% serum challenge. Both anacardic acid (30 µM) and trichostatin (0.5 and 1 µM) significantly attenuated serum-induced proliferation of H2691 mesothelioma cells; however, had minimal effect on control Met5A cells (**Fig. 7A and 7B**). These results suggested that inhibition of both HATs and HDACs attenuate mesothelioma cell proliferation by serum.

### Knockdown of SphK1 Regulates Expression of p300 (CBP), PCAF, p21Cip1 and p27Kip1 in Mesothelioma Cells

Having demonstrated a role for SphKs in histone acetylation and phosphorylation, we investigated the role of SphK1 on expression of histone acetyl transferases (HATs). Down-regulation of SphK1 with specific siRNA for 48 h (>80% loss in specific protein expression, data not shown) attenuated mRNA levels of CBP and p300/CBP-associated factor (PCAF) in mesothelioma cell line H2691, but not the control Met5A cells (**Fig. 8A and 8B**). However, the expression of another HAT, MYST2 was not affected by SphK1 siRNA in both Met5A and H2691 cells (**Fig. 8C**). CBP has been linked to p27Kip1 transcription [35]. We therefore investigated the effect of SphK inhibition on expression profile of two members of the Cip/Kip family of cell cycle inhibiting cyclin-dependent kinases. As shown in **Fig. 8D** **,** pretreatment of with the SphK inhibitor, SPHK-I_2_ (10 µM) for 24 h, increased the expression of both p21Cip1 (∼7 fold) and p27Kip1 (∼3 fold) in H2691, but not Met5A, as determined by quantitative real time RT-PCR using specific gene primers. These results suggest a role for SphK1 in modulating expression of HATs in mesothelioma cells.

### SphK Modulates Genes Involved in Chromatin Remodeling and Promoter Activity

To further support the role of SphK1 in regulating mesothelioma cell proliferation by chromatin modification of targeted gene promoters, we examined the effect of SphK inhibitor on histone acetylation of Transcriptional Start Site (TSS; minimal promoter) regions of cell cycle related genes using chromatin immunoprecipitation (ChIP) with acetyl lysine antibody. Enriched DNA was subjected to PCR amplification with gene specific primers as described in methods and material section. As shown in **Fig. 9A** **,** SphK inhibitor treated H2691 cells showed decreased promoter histone acetylation of AKB (aurora kinas B, (involved in chromosome segregation and targeted for cancer therapy); Top2A (the DNA unwinding enzyme Topoisomerase 2 alpha); but not that Of HAT1, a known histone acetyltransferase that is elevated in several cancers, and MYST2 [33]. No change was observed in Met5A cells suggesting that these changes were specific to MPM cells and was regulated by SphK1. In contrast, an increase in acetylation was observed p27KiP1 and p21CiP1, when H2691 but not Met5A cells were treated with the SphK-I_2_ (**Fig. 9B**), which is in complete agreement with respect to their transcript levels shown in **Fig. 8D**. The relative changes can be appreciated in the relative densities of the bands shown as bar graphs in **Fig. 9** **B** and **9C**.

### Multi Walled Nanotubes-induced Granulomatous Inflammation is Attenuated in *SphK1^−/−^* Mice

To test our hypothesis that SphK1 is an effective novel therapeutic target in malignant mesothelioma, we investigated multi walled nanotubes (MWNT)-induced granulamatous inflammation in control and *SphK1^−/−^* mice [33]. Male *SphK1^−/−^* mice and their wild type counterparts were injected with a single dose of 50 µg or 3 mg of MWNT into intra-peritoneal cavity. After 12 days, abdominal wall was dissected exposing the peritoneal cavity, and the diaphragm was carefully removed and analyzed as described in Methods. Hematoxylin and eosin stained histology sections show the presence of dose dependent increase in granulamatous inflammation (early mesothelioma like symptom) in C57BL/6 mice which were exposed to MWNT, compared to PBS injected mice (**Fig. 10** **a–c**). SphK1 null mice diaphragm sections on the other hand exposed to MWNT exhibited decreased granulamatous inflammation compared to control mice injected with MWNT (**Fig. 10** **d–f**). Semi-quantification of granulamatous inflammation by Image analyzer confirmed attenuation of MWNT-mediated inflammation in SphK1 deficient mouse [Relative area of the granulomas (mm^2^): wild type mice- sham,0; 50 µg, 19055; 3 mg, 38197; SphK1^−/−^ mice – sham, 0; 50 µg, 4483; 3 mg, 15988].

## Discussion

Malignant mesothelioma is an aggressive neoplasm that arises from the serosal surfaces of pleura, or peritoneum or pericardium. Prolonged exposure to asbestos is a well-known risk factor [1]. Therapy involves surgery combined with radiation and/or chemotherapy. To make a dramatic impact on the overall survival rate in mesothelioma, there is an immediate urgency for the development of newer targeted, highly efficacious therapies. Recently we have shown that VEGFR/PKC-β2/AKT signal transduction to be actively involved and essential for the survival of malignant pleural mesothelioma [36]. In the present study, we demonstrate a definitive role for SphK1, but not SphK2, in supporting the proliferation of mesothelioma cells. The underlying mechanism appeared to be mediated by SphK1 induced promotion of select HAT activities such as HAT1, CBP/p300 and PCAF. The resulting effect is the suppression of cell cycle dependent kinase inhibitor genes such as p21Cip1 and p27Kip1, and activation of other cell proliferation related genes such as HAT1 (Histone acetyl transferase), Top2A (DNA replication), AKB (chromosome remodeling), and CDK2 (cell cycle check point regulator) as deduced from ChIP assay. Finally, using a mouse model, we demonstrated that abrogation of SphK1 resulted in significantly reduced MWNT-induced peritoneal granulomas.

SphK1 and SphK2 have both been implicated in tumorigenesis; however, there is much less evidence for SphK2 involvement in cancer. SphK1 enhances foci and growth in soft agar and its overexpression in pro-erythroblasts conferred tumorigenecity in vivo [37]–[39] despite the fact that no oncogenic mutant forms of SphK1 have been detected. It is also highly expressed in a variety of cancers [17] such as head and neck [40], [41], prostate [42], colon [43], breast [44], glioma [45]–[47], salivary [48], non-small cell lung [49], and gastric cancers [50]. On the other hand, knockdown of SphK2, but not SphK1, in U1242 and U87 MG glioblastoma cells attenuated cell proliferation and doxorubicin-induced apoptosis in MCF7 breast cancer cells [51], [52]. Further, SphK2, in addition to SphK1, plays an important role in migration of MDA-MB-453 cells towards EGF [28]. In this study, we have demonstrated for the first time, relatively higher protein expression of SphK1, but not SphK2, in mesothelioma cell lines. In contrast to the protein expression, mRNA levels of both SphK1 and SphK2 were higher in mesothelioma cell lines compared to control Met5A. Protein expression is dictated by several factors, including level of mRNA half life, translation, and turnover rate of the protein of interest. Also, it is evident that there is no direct correlation between mRNA expression levels and protein expression, and in many instances, an increase in mRNA expression does not necessarily translate to similar level of protein expression [53], [54]. Thus, our current observation on mRNA and protein expression of SphK1 and SphK2 are in accordance with reports in the literature with respect to no direct correlation between mRNA and protein expression.

SphK1 rather than SphK2 appears to be to be over-expressed in several cancers and our finding in MPM is also in agreement with the above. In addition, several studies have attested to the cell proliferation promoting and apoptosis preventing ability of SphK1 [17], [44]–[47]. Our studies using SphK-I_2_ and siRNA specific for SphK1 are in accordance (**Fig. 3** and **Fig. 4**). Although the levels of SphK1 are detectable in Met5A control cells, knocking down SphK1 in these cells had no discernable effect on cell proliferation thereby suggesting that SphK1’s growth promoting role may be specific to mesothelioma cancer cells. In addition to expression, it was also reported that there was increased enzymatic activity of SphK1 in prostate cancer that directly correlated with the higher PSA expression [42]. In the present study, we also observed formation of intracellular S1P, the product generated by SphK1 activity under basal conditions; however, the ability to generate intracellular S1P when challenged with its substrate sphingosine was much higher in mesothelioma cell lines (>3X) compared to Met5A control cells (**Fig. 2**) that can be attributed to the significant amounts of SphK1 expressed (**Fig. 1**). Despite this observation, addition of S1P to serum free media had either inhibitory or no effect on Met5A controls whereas there was a small dose dependent increase in cell proliferation of H2691 cells (data not shown). This raises the possibility that there may be two pathways involved in SphK1 dependent mesothelioma cell proliferation. One pathway is S1P dependent that requires SphK1 and/or SphK2 activity and the other pathway is SphK1 dependent but S1P independent.

Further investigation into the mechanism underlying the role of SphK1 in mesothelial cell proliferation revealed that abrogation of SpkK1 expression results in a dramatic loss in CDK2 phosphorylation (**Fig. 5**). As CDK2 activity is required for early cell cycle progression, it is not surprising that DNA replication is arrested in SphK1 knockdown H2691 cells. In order to further understand the mechanism behind the increased CDK2 phosphorylation in H291 cells, we determined the relative levels of CDK inhibitor transcripts p21Cip1 and p27Kip1. Complementing the above, we found elevated transcript levels of p21Cip1 and p27Kip1 in response to SphK-I_2_ treatment in H2691 but not in Met5A cells (**Fig. 9**). This is in complete agreement with observed inhibition of mesothelioma cell proliferation when treated with SphK-I_2_. Radiation is known to arrest cell proliferation and induce apoptosis in cancer cells; however in a study using malignant mesothelioma cell line exposed to radiation, it was observed that there was no change in p27Kip1 levels thus making SphK1 mediated signaling pathway in mesothelioma cells unique [55]. Furthermore, our results concur with an earlier finding demonstrating a causal link between p27Kip1 degradation due to its phosphorylation and degradation in cells that are not in G1 phase [56]. In addition to p21Cip1 and p27Kip1, arrest in G1 phase is also dependent on p53 expression [57]; however, the role of p53 in SphK1 inhibited mesothelioma cell proliferation needs to be investigated. Also, in MCF7 cells SphK2 dependent p27Kip1 expression induced by doxorubicin treatment was found to be p53-independent suggesting different mechanism(s) underlying SphK1 and Sphk2 mediated signals [51]. It also must be pointed out that transcriptional induction of p21Cip1 and p27Kip1 mRNA by SphK inhibitor in mesothelioma cells did not translate to increased protein levels (**Fig. 9**) thereby questioning the importance of CDK inhibitors in SphK1 mediated signaling in mesothelioma cells.

The state of chromatin structure determines whether a particular gene is expressed or not. The human genome in the cell is packaged into chromatin that is a dynamic macromolecular complex consisting of DNA, histone and non-histone proteins. The chromatin consists of repeating units known as nucleosome. The DNA in the nucleosome is wrapped around by histone octamer that is formed by H3 and H4 tetramer and two H2A and H2B dimmers. H1 is wrapped around the inter-nucleosomal linker DNA, The open chromatin structure favoring gene transcription is facilitated by acetylation of H3 and H4 histones [4]. Another important finding made in the present study is the role played by SphK1 in the acetylation of H3 and H4 histones in mesothelioma cells. Although, earlier studies have shown that SphK1 is involved in tumor growth and cancer cell proliferation [16], we provide evidence for the first time that SphK1-mediated histone acetylation as a necessary step in regulating mesothelioma cell proliferation. Histone acetylation, (post-translational addition of acetyl groups to lysine residues) reduces the positive charge on core histone and decreases the affinity of histone to DNA [58]. This loosening of histone to DNA facilitates accessibility of transcriptional factors to promoter regions of genes [59]. Core histone acetylation is a reversible process regulated by histone acetyl transferases and histone deacetylases [59]. We demonstrated that both H3 and H4 histone acetylation that are enhanced in the presence of serum are significantly suppressed upon the addition of SphK-I_2_ clearly supporting our contention that SphK1 mediated signaling triggers gene transcription programs that support cell survival and proliferation by regulating histone acetylation. We also demonstrated specificity by clearly showing that abrogation of SphK1 in H2691 cell resulted in marked reduction in p300 (CBP) and PCAF transcripts but not that of MYST2 (**Fig. 8**). In addition, using the ChIP assay, we also demonstrated that prior treatment of H2691 cells with SphK-I_2_ resulted significant drop in association between acetylated histone and specific gene promoters such as HAT1, CDK2, TOP2A and AKB (**Fig. 9**). Our results thus support the idea that SphK1 signaling in mesothelioma cells may recruit specific HATs. In contrast to HATs, histone deacetylases (HDAC) suppress gene transcription by removing the acetyl group from histone. HDAC inhibitors prevent deacetylation of histones and thus favor gene transcription. Despite the above, several HDAC inhibitors are currently in various stages of cancer clinical trials. It is believed that these drugs preferentially suppress deacetylation of several key tumor suppressor promoters that includes CDK inhibitors such as p21Cip1 and p27Kip1 [4]. This could explain the observed inhibitory effect of the HDAC inhibitor, TSA on mesothelioma cell proliferation (**Fig. 7**). Interestingly, in breast cancer MCF-7 cells, SphK2, but not SphK1, was associated with histone H3 and on stimulation with phorbol ester produced S1P [32], [60]. Further, the S1P generated in the nucleus via SphK2 was associated with HDAC1 and HDAC2 complex, inhibited HDAC activity and was selectively enriched at the promoter of *CDKN1A* (encoding for p21) and *C-FOS* [59]. Although intracellular generation of S1P is higher in mesothelioma cells, they hardly express SphK2. It is possible that SphK1 in conjunction with S1P many have a similar role to that of SphK2 in modulating HDACs in mesothelioma, which is currently under investigation. This is also supported by the fact that some of the HDAC inhibitors are currently at various stages of cancer clinical trials including MPM [1].

Finally, due to a lack of proper mouse model to investigate mesothelioma, using SphK1 knockout mice, we demonstrated a clear reduction in peritoneal granulamatous tissue as compared to their wild type counterparts when challenged with intra peritoneal injection of multi-walled carbon nanotubes. It was recently shown that exposing the mesothelial lining of the body cavity of mice, as a surrogate for the mesothelial lining of the chest cavity, to long multiwalled carbon nanotubes results in the development of granulomas in p53 knockout mice [33]. Here, we observed inflamed granulamatous tissue lining the diaphragm of mice exposed to MWNT that was significantly suppressed in SphK1 knockout mice (**Fig. 10**), which supports a role for SphK1 in tumor promotion [39]. However, our results do not exclude the involvement of SphK2 in mesothelioma and further studies are necessary to understand whether both isoforms play a role in mesothelioma. Here, we propose that SphK1 in is an ideal target candidate in the development of mesothelioma therapeutics.
